# A retrospective cohort study in patients with tractional diseases of the vitreomacular interface (ReCoVit)

**DOI:** 10.1007/s00417-016-3294-1

**Published:** 2016-02-22

**Authors:** Peter Stalmans

**Affiliations:** Department of Ophthalmology, University Hospitals Leuven, Herestraat 49, 3000 Leuven, België Belgium

**Keywords:** Retina, Macula, Macular hole, Vitreomacular adhesion, Vitreomacular interface, Vitreomacular traction

## Abstract

**Purpose:**

To assess how vitreomacular adhesion (VMA), vitreomacular traction (VMT), and macular holes (MH) evolve, and to assess visual acuity outcomes associated with different management strategies for each subgroup.

**Methods:**

Retrospective, single-center, observational study of 400 patients (556 eyes) who presented with optical coherence tomography (OCT) findings related to tractional diseases of the vitreomacular interface (187 with bilateral disease). The outcomes measured include prevalence of symptoms, rates of disease stabilization, spontaneous resolution, and disease progression necessitating surgical intervention. Size of VMA/VMT was not measured.

**Results:**

Vision loss and metamorphopsia were the leading causes for referral. Patients were followed for a mean of 10.9 months (median 6.9 months). Spontaneous resolution occurred in 22.7 % (46/203) of eyes with VMT and in 7.3 % (9/124) of eyes with VMA (*P* < .001). In the former group, 34.1 % (14 eyes) showed improved visual acuity (P = .001). During follow-up, 11.3 % (14/124) of eyes with VMA showed disease progression; six (4.8 %) developed a macular hole. Eleven of the 203 eyes with VMT (5.4 %) developed a macular hole; 52 of 203 eyes with VMT (25.6 %) had disease progression that resulted in patients opting for pars plana vitrectomy (PPV). Of the eyes with VMA, 4.8 % (6/124) had disease progression resulting in patients opting for PPV.

**Conclusions:**

Better visual acuity outcomes were found in eyes with spontaneous resolution compared to the other groups. Spontaneous resolution of VMT and VMA was rare, whereas disease progression resulting in PPV was more common.

## Introduction

Posterior vitreous detachment (PVD) is a consequence of aging whereby the vitreous liquefies and detaches from the internal limiting lamina of the retina [1, 2]. When the liquefaction exceeds vitreoretinal dehiscence and traction is exerted at the vitreomacular interface (VMI), an anomalous PVD occurs [1].

Numerous potential anatomic consequences of anomalous PVD exist. Forced tractional separation (either surgical or mechanical) between the posterior vitreous and the internal limiting membrane (ILM) has shown pockets of extreme adhesion; subsequent methods to break the bond can result in retinal tears and dialyses [2]. Other potential anatomic effects include vitreomacular adhesion (VMA) and vitreomacular traction (VMT) syndromes or hemorrhage in the presence of concomitant disease [1]. Focal VMT can result in macular hole (MH) formation or tractional cystoid macular edema, while broad VMT has been associated with epiretinal membrane (ERM) and diffuse retinal thickening, among others [3]. However, the progression of VMA and VMT is not consistent among all patients, and data on the natural history are limited.

Visual acuity (VA) is usually preserved in early VMT, but is progressively impacted as a macular hole develops [4]. VMT can also lead to visual disturbances, among them metamorphopsia, photopsia, and micropsia [5–11]. There are very limited data on the impact of these symptoms on visual quality in patients with VMT; however, metamorphopsia has been associated with decreased vision-related quality of life in other vitreoretinal disorders [5, 6, 12–15], and it has been suggested that it may be more important to overall perception of visual function than visual acuity [13].

In patients with tractional VMI disease, “watchful waiting” has been the traditional standard of care. Pars plana vitrectomy (PPV) is typically reserved for more severe or progressive cases [6, 16]. In some patients, watchful waiting results in favorable outcomes, including spontaneous resolution of VMA [10]. However, anatomic improvements do not necessarily correlate with visual function (VA or symptoms) [10], and PPV has been reported to have a relatively modest impact on VA [17].

Epidemiological data on VMA, VMT, and MH also are limited [18–22]. In today’s real-world clinical settings, a confirmed diagnosis on spectral-domain OCT (SD-OCT) has made it much easier for clinicians to examine the vitreoretinal interface and identify tractional diseases, often before visual loss becomes irreversible [10, 11, 23, 24]. However, since the incorporation of SD-OCT, few large-scale studies have been published.

The goal of this retrospective analysis of electronic medical records was to both extract data on VMA, VMT, and MH from a clinical setting, and to assess how the condition in each subgroup evolves. We planned to determine patient characteristics at baseline, the prevalence of symptoms, and the clinical course of the disease in the prepharmacologic vitreolysis era, including the incidence of spontaneous resolution, PPV, and disease progression, as well as the impact of treatment practices on VA outcomes. Our analysis represents the largest observational cohort to date that includes patient follow-up and with a confirmed diagnosis of tractional disease of VMI using SD-OCT imaging [10, 22, 25–36].

## Methods

This was a retrospective, single-center, observational study conducted with patients attending in the ophthalmology unit of the University Hospitals Leuven (Universitaire ziekenhuizen Leuven [UZLeuven]), a large tertiary care center in Flanders, Belgium. The time frame for this study encompassed the period before a pharmacologic treatment for VMT was commercially available. At that time, the standard of care at our center was to recommend PPV for patients presenting with a macular hole. Patients with VMA or VMT were predominantly managed by watchful waiting until disease progression reached a threshold where the patient opted for surgical intervention.

### Inclusion criteria

**Fig. 1:**
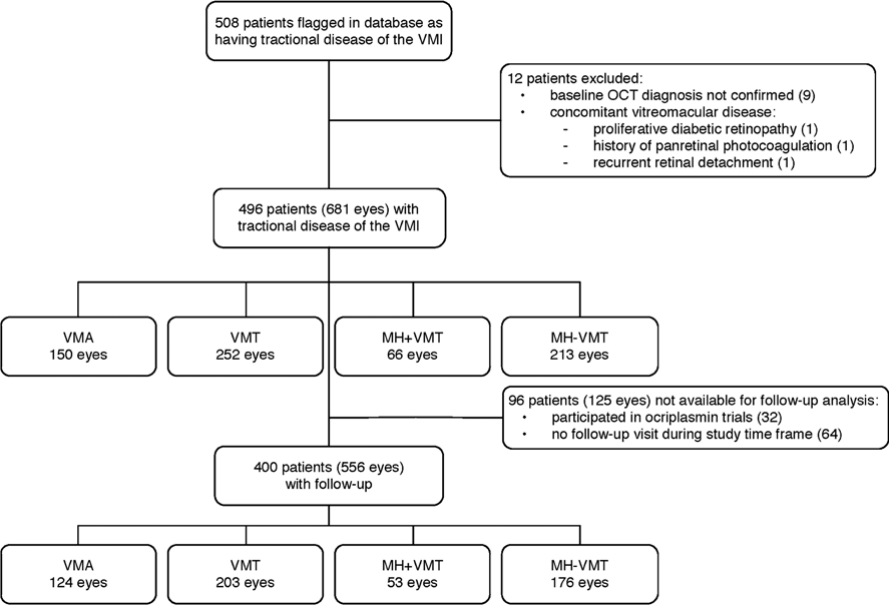
Patient disposition flow diagram (CONSORT). Flow diagram depicting the progress and disposition of patients, from identification in the database through analysis and inclusion/exclusion into dataset

Consecutive patients presenting at the center between July 2009 and August 2013 were included if they had SD-OCT findings related to tractional diseases of the VMI (as recently classified by Duker et al.) [37]. Both symptomatic and asymptomatic patients were included. For patient allocation, our hospital has developed subcodes to the ICD coding system specifically for MH and VMA. These subcodes were used to locate all patients in the database since 2009 that had these specific ocular conditions, and this information was used to develop the consort diagram represented in Fig. 1.

### Exclusion criteria

Patients were excluded from the study if there was concomitant retinal pathology potentially influencing VA during follow-up (ie, proliferative diabetic retinopathy, history of panretinal photocoagulation, or recurrent retinal detachment), had isolated ERM without VMA or VMT (ie, isolated pucker formation), or if they participated in clinical trials using ocriplasmin. Broad attachments were not excluded. VMT with concomitant ERM was defined as two separate layers that could be distinguished on OCT, one being the posterior hyaloid membrane and one being the epiretinal membrane.

### Technique

All surgeries were 23-gauge transconjunctival vitrectomies, with internal limiting membrane (ILM) peel performed in all cases after staining with ILM-Blue (DORC, Netherlands). With respect to lens status, the recommended treatment algorithm of a combined phaco-vitrectomy was performed for all consenting phakic patients over 50 years of age. In these cases, lens surgery was performed first, followed by vitrectomy. All surgeries for macular hole involved the use of silicone oil, where a second surgery was performed 3 months after the first surgery to remove the oil. Silicone oil was used due to its increased density (heavier than water), providing a good tamponade of both the inferior and posterior pole in normal head posturing, obviating the need for postoperative face-down positioning [38–40].

### Data collection and analysis

Raw clinical and OCT data (Cirrus OCT, Carl Zeiss Meditec, Jena, Germany) were extracted from the electronic medical records system used in the tertiary care center (Klinisch WerkStation [Clinical Workstation], developed at UZLeuven, Belgium). Data analysis was performed by an independent statistician.

VA was collected in decimal format and converted to logMAR for statistical analysis. A VA change of ≥0.2 logMAR units (corresponding to two ETDRS lines) was deemed to be clinically meaningful.

For SD-OCT, a 5-series 6-mm line scan was performed over the macular area, as well as a 512 × 128 macular cube scan. SD-OCTs were examined from both eyes. All eyes with tractional pathology of the VMI were included and classified according to the recently published International Vitreomacular Traction Study Group (IVTS) classification in four groups with increasing disease stage: VMA, VMT, MH with VMT, and MH without VMT [37]. Size of VMA/VMT was not measured. In cases of bilateral involvement, the eye with worse pathology was considered to be the index eye. If both eyes had the same disease stage, the eye with worst visual acuity was considered to be the index eye.

Collected baseline data included age, gender, and duration of symptoms prior to baseline visit, source, and reason for referral, VA, presence of metamorphopsia, concomitant ERM, and history of diabetes. The outcomes of interest were evolution of visual acuity, incidence of PPV, spontaneous resolution of traction, and progression of disease. Clinically meaningful changes in visual acuity were defined as changes equivalent to two or more ETDRS lines. Visual acuity measurements less than 30 days after PPV were excluded from analyses. Kaplan-Meier analysis was used for cumulative incidence curves representing time to PPV and spontaneous resolution. Nonparametric tests were used for comparisons of vitreomacular disease groups. The Wilcoxon matched-pairs signed-ranks test was used to evaluate the evolution of VA over time. VA outcomes in VMT patients were compared across outcomes (PPV, spontaneous resolution, continued watchful waiting).

A logistic regression model was fit with potential baseline predictors for vitrectomy in VMT eyes only. Age, gender, metamorphopsia, VA, and duration of symptoms prior to diagnosis were included. Nonsignificant (P ≥ .05) baseline variables were stepwise dropped from the model until all remaining predictors were significant. Statistical analysis was performed using Stata version 13.1 for Mac (StataCorp LP, TX, USA).

The study protocol and investigators for the site were approved by the ethics committee of the UZLeuven.

## Results

In total, 496 patients with 681 affected eyes were included for baseline analysis. Of those, 400 patients (556 eyes) had at least one follow-up visit (See Fig. 1 for patient disposition). There were no differences in demographic characteristics or distribution of tractional vitreomacular disease between those with or without follow-up. Women made up two-thirds of the cohort. Following the classification of the International Vitreomacular Traction Study Group [37], at baseline, the majority of eyes were diagnosed with VMT (37.0 %), followed by MH without VMT (31.3 %), VMA (22 %), and finally MH with VMT (9.7 %). In patients with follow-up data, the distribution among groups was similar: VMT (36.5 %), MH without VMT (31.7 %), VMA (22.3 %), and MH with VMT (31.7 %) (Table 1). Therefore, the distribution of patients with at least one follow-up visit was representative of the whole patient population. These patients were followed for a mean of 10.9 months (median 6.9 months).

**Table 1:** Baseline demographics, all eyes with follow-up (N = 556)

	VMA (N = 124)	VMT (N = 203)	MH + VMT (N = 53)	MH–VMT (N = 176)	All Eyes (N = 556)
Age, years, mean (SD)	68.9 (9.22)	72.6 (8.99)	68.7 (6.49)	68.7 (10.05)	70.2 (9.35)
Male: n (%)	38 (30.7)	82 (40.4)	10 (18.9)	57 (32.4)	187 (33.6)
Duration of symptoms prior to diagnosis, mo, median (Q1-Q3)	2.8 (0.82–3.73)	2.8 (1.87-4.67)	1.9 (0.70-3.27)	2.8 (1.87-5.60)	2.8 (1.87-5.60)
Follow-up (months): median (Q1-Q3)	6.8 (5.15-14.90)	8.1 (3.73-16.80)	6.4 (5.27-11.80)	6.5 (5.13-11.80)	6.9 (4.90-14.40)
Reason for referral:					
Vision loss (far), n (%)	15 (50.0)	100 (54.6)	25 (48.1)	99 (60.7)	239 (55.8)
Vision loss (near), n (%)	14 (46.7)	63 (34.4)	17 (32.7)	49 (30.1)	143 (33.4)
Any vision loss, n (%)	17 (56.7)	112 (61.2)	32 (61.5)	104 (63.8)	265 (61.9)
Metamorphopsia, n (%)	7 (23.3)	45 (24.6)	29 (55.8)	67 (41.1)	148 (34.6)
Baseline characteristics:					
logMAR, mean (SD)	0.11 (0.196)	0.35 (0.310)	0.72 (0.348)	0.86 (0.330)	0.49 (0.422)
Metamorphopsia: n (%)	22 (17.7)	102 (50.3)	49 (92.5)	146 (83.0)	319 (57.4)
ERM, n (%)	4 (3.2)	28 (13.8)	0 (0.0)	1 (0.6)	33 (5.9)
Diabetes, n (%)	10 (8.1)	26 (12.8)	2 (3.8)	27 (15.3)	65 (11.7)

*ERM*, epiretinal membrane; *logMAR*, logarithm of the minimum angle of resolution; *MH + VMT*, macular hole with vitreomacular traction; *MH-VMT*, macular hole without vitreomacular traction; *SD*, standard deviation; *VMA*, vitreomacular adhesion; *VMT*, vitreomacular traction.

### Disease characteristics

A total of 496 patients had a bilateral evaluation at initial presentation, of which 185 patients (37.3 %) had bilateral tractional VMI disease (Table 2).

**Table 2:** Fellow eye disease status in relation to index eye disease status

	Index eye vitreomacular disease stage				
Fellow eye vitreomacular disease status	VMA	VMT	MH + VMT	MH-VMT	Total
No vitreomacular disease	25 (69.4)	116 (61.1)	33 (54.1)	137 (65.6)	311 (62.7)
VMA	11 (30.6)	35 (18.4)	15 (24.6)	53 (25.4)	114 (23.0)
VMT		39 (20.5)	11 (18.0)	12 (5.7)	62 (12.5)
MH + VMT			2 (3.3)	3 (1.4)	5 (1.0)
MH-VMT				4 (1.9)	4 (0.8)
Total	36 (100.0)	190 (100.0)	61 (100.0)	209 (100.0)	496 (100.0)

*ERM*, epiretinal membrane; *MH + VMT*; macular hole with vitreomacular traction; *MH-VMT*, macular hole without vitreomacular traction; *SD*, standard deviation; *VMA*, vitreomacular adhesion; *VMT*, vitreomacular traction.

**Fig. 2:**
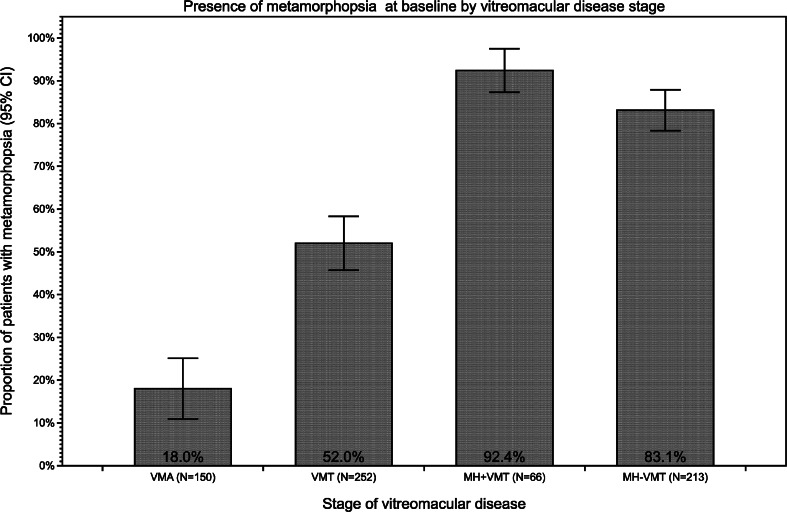
Metamorphopsia at baseline by stage of VMI disease (N = 681). Graph representing the proportion of patients at baseline with metamorphosia at different stages of vitreomacular disease (VMA, VMT, MH + VMT, MH-VMT). CI, confidence interval; MH + VMT, macular hole with vitreomacular traction; MH-VMT, macular hole without vitreomacular traction; VMA, vitreomacular adhesion; VMI, vitreomacular interface; VMT, vitreomacular traction

**Fig. 3:**
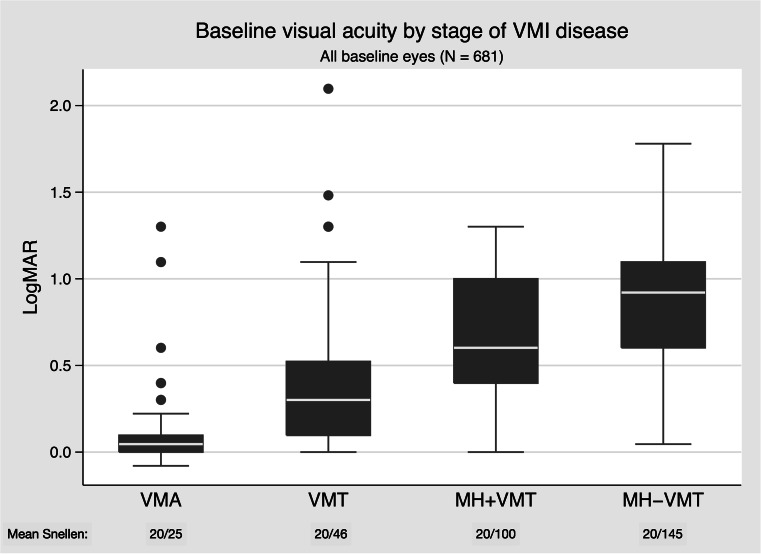
Baseline visual acuity by stage of VMI disease. Boxplot depicting baseline visual acuity for patients with different stages of tractional VMI disease. logMAR, logarithm of the minimum angle of resolution; MH + VMT, macular hole with vitreomacular traction; MH-VMT, macular hole without vitreomacular traction; VMA, vitreomacular adhesion; VMI, vitreomacular interface; VMT, vitreomacular traction

The proportion of eyes with metamorphopsia at baseline was highest in eyes with MH with or without VMT (92.4 % and 83.1 %, respectively), followed by those with VMT (52 %). However, even in the VMA group, 27/150 (18.0 %) had symptoms of metamorphopsia, despite a lack of tractional abnormalities on OCT (Fig. 2). The boxplot in Fig. 3 shows how baseline VA progressively worsened with advancing stage of VMI disease (nonparametric test for trend: *P* < .001).

### Patient outcomes

Table 3 describes the overall patient outcomes; almost half the patients underwent PPV.

**Table 3:** Outcomes in eyes with at least one follow-up visit (N = 556).

	VMA (N = 124)	VMT (N = 203)	MH + VMT (N = 53)	MH-VMT (N = 176)	All Eyes (N = 556)
Baseline visual acuity: N	124	198	53	176	551
LogMAR, mean (SD)	0.11 (0.196)	0.35 (0.310)	0.72 (0.348)	0.86 (0.330)	0.49 (0.422)
Mean Snellen	20/25	20/45	20/105	20/145	20/61
Last observed visual acuity: N	124	193	49	171	537
LogMAR, mean (SD)	0.14 (0.248)	0.32 (0.366)	0.56 (0.444)	0.67 (0.391)	0.41 (0.414)
Mean Snellen	20/28	20/42	20/73	20/94	20/51
Spontaneous resolution, n (%)	9 (7.3)	46 (22.7)	N/A	N/A	n/a
Vitrectomy, n (%)	6 (4.8)	52 (25.6)	47 (88.7)	152 (86.4)	257 (46.2)
Progression, n (%)	14 (11.3)	11 (5.4)	8 (15.1)	N/A	N/A
Progression to MH, n (%)	6 (4.8)	11 (5.4)	N/A	N/A	N/A

*logMAR*, logarithm of the minimum angle of resolution; *MH + VMT*, macular hole with vitreomacular traction; *MH-VMT*, macular hole without vitreomacular traction; *N/A*, not applicable; *SD*, standard deviation; *VMA*, vitreomacular adhesion; *VMT*, vitreomacular traction

#### Disease progression

**Fig. 4:**
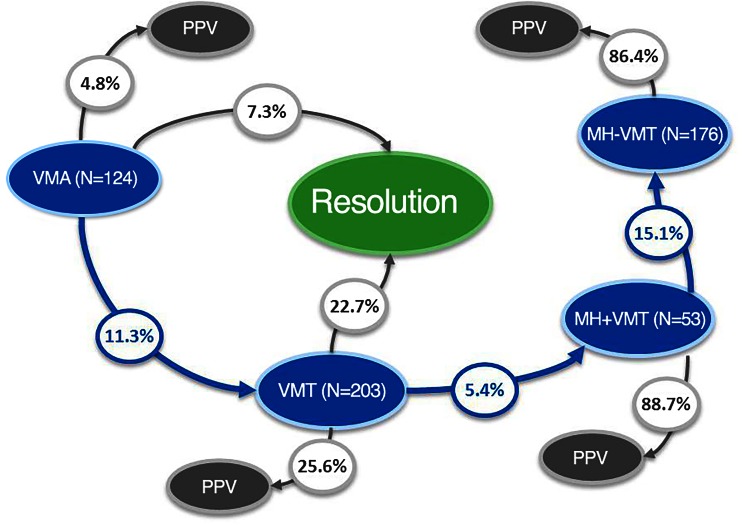
Flow chart on patient outcomes by baseline disease. Flow diagram depicting proportion of patients progressing through different stages of VMI disease and treatment. Patients were followed for a mean of 10.9 months (median 6.9 months). MH + VMT, macular hole with vitreomacular traction; MH-VMT, macular hole without vitreomacular traction; PPV, pars plana vitrectomy; VMA, vitreomacular adhesion; VMI, vitreomacular interface; VMT, vitreomacular traction

During follow-up, 11.3 % (14/124) of eyes with VMA showed disease progression, with six eyes (4.8 %) developing a macular hole. Of the 203 eyes with VMT, 11 (5.4 %) developed a macular hole; five of these (45.5 %) were within the first month. Within the “MH with VMT” group, 15.1 % (8/53) of eyes evolved toward detachment; none of the macular holes closed spontaneously (Fig. 4).

#### Spontaneous resolution

**Fig. 5:**
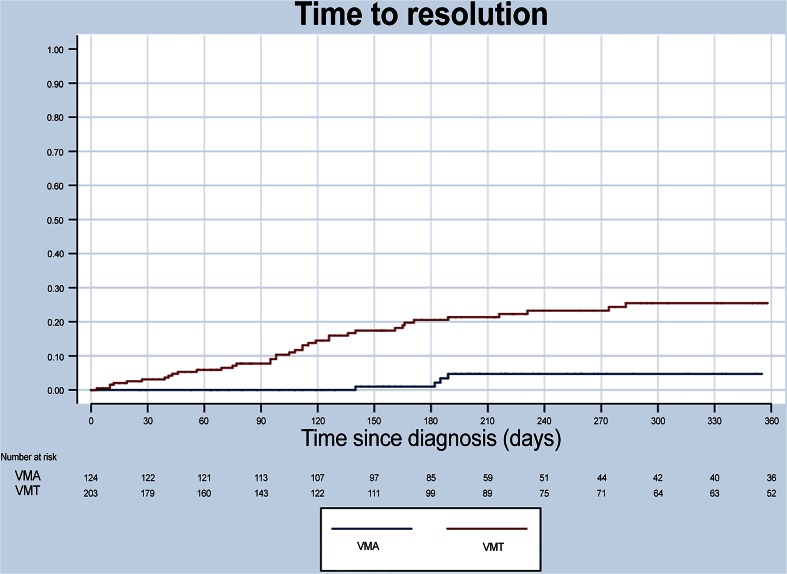
Time to resolution in eyes with VMT or VMA. Kaplan Meyer graph showing the proportion of patients with VMT or VMA at baseline that achieved spontaneous resolution within 1 year of diagnosis. VMA, vitreomacular adhesion; VMT, vitreomacular traction

Eyes with VMT were more likely to experience spontaneous resolution of traction than eyes with VMA (Fig. 5): 22.7 % (46/203) of eyes vs. 7.3 % (9/124) of eyes, respectively, within 1 year after diagnosis (Fisher’s exact test; *P* < .001).

#### Vitrectomy

**Fig. 6:**
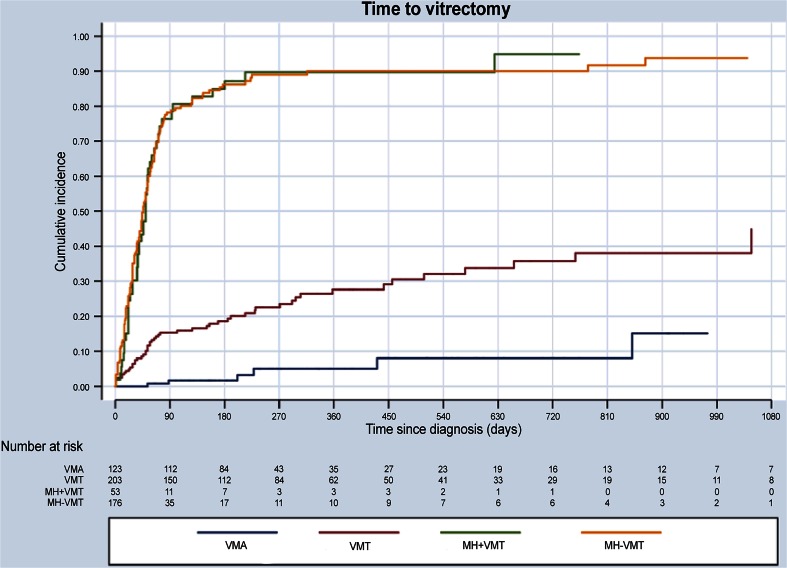
Time to PPV by VMI disease stage. Kaplan Meyer graph showing the proportion of patients with VMA, VMT, MH + VMT, and MH-VMT who had PPV within 1 year of diagnosis. MH + VMT, macular hole with vitreomacular traction; MH-VMT, macular hole without vitreomacular traction; VMA, vitreomacular adhesion; VMT, vitreomacular traction; VMI, vitreomacular interface

In the “MH with VMT” group, 88.7 % (47/53) of eyes underwent PPV, similar to the 86.4 % (152/176) of eyes in the “MH without VMT” group. Although most eyes with VMT were managed by watchful waiting, 25.6 % (52/203) of VMT eyes underwent PPV during follow-up, mostly because their diseased worsened. Even in the VMA group, 4.8 % (6/124) of eyes underwent vitrectomy, four because of disease-stage progression (Fig. 6).

#### Visual acuity outcomes

Table 4 shows the change in VA from baseline to last observation. Visual acuity outcomes differed depending on the stage of VMI disease. More than 50 % of the 220 eyes with a macular hole at baseline had an improved VA at their last visual assessment. In eyes with a macular hole that underwent PPV, 57.9 % (110/190) had VA improvement, while VA in the 30 eyes with a macular hole that did not undergo PPV remained stable (18 eyes, 60.0 %) or worsened (six eyes, 20 %; Fisher’s exact test, *P* < .001). Nevertheless, 13.2 % (13/190) of eyes with a macular hole that underwent PPV experienced worsening VA. In eyes affected by VMA or VMT, VA remained largely stable during follow-up.

**Table 4:** Clinically meaningful change in visual acuity by stage of VMI disease

≥ 0.2 logMAR change	VMA	VMT	MH + VMT	MH-VMT	Total
Worse: n (%)	9 (7.3)	16 (8.5)	9 (18.4)	22 (12.9)	56 (10.3)
Unchanged, n (%)	110 (88.7)	133 (70.7)	14 (28.6)	59 ( 4.5)	316 (9.4)
Improved: n (%)	5 (4.0)	39 (20.7)	26 (53.1)	90 (52.6)	160 (30.1)
Total: (%)	124 (100.0)	188 (100.0)	49 (100.0)	171 (100.0)	532 (100.0)
Change, median (Q1, Q3)	0.0 (0.00, 0.05)	0.0 (−0.18, 0.07)	−0.3 (−0.48, 0.00)	−0.2 (−0.48, 0.00)	0.0 (−0.26, 0.05)

*logMAR*, logarithm of the minimum angle of resolution; *MH + VMT*, macular hole with vitreomacular traction; *MH-VMT*, macular hole without vitreomacular traction; *Q1*, interquartile 1; *Q3*, interquartile 3; *VMA*, vitreomacular adhesion; *VMI*, vitreomacular interface; *VMT*, vitreomacular traction.

#### VA outcomes in eyes with VMT

**Fig. 7:**
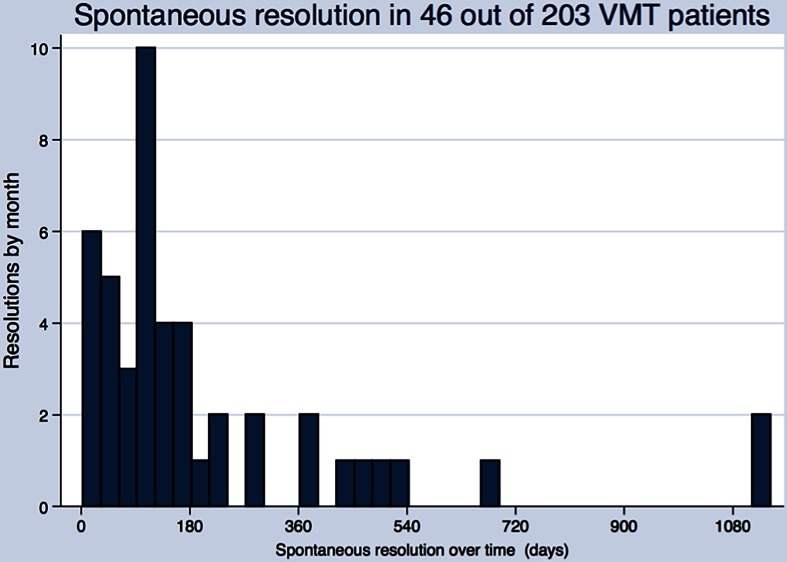
Time to spontaneous resolution in eyes with VMT. Bar graph showing number of eyes in the VMT group with spontaneous resolution over time (days). Each bar represents the number of resolutions per month. VMT, vitreomacular traction

**Fig. 8:**
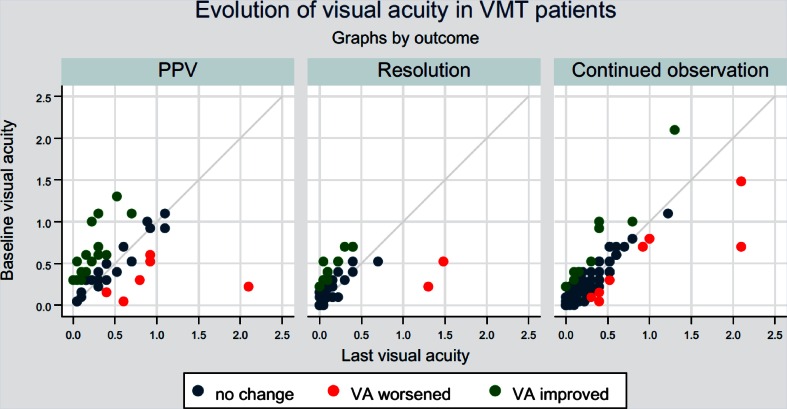
Evolution of visual acuity in patients with VMT. Scatter plot of the evolution of visual acuity in VMT patients by outcome, illustrating change in VA (improved, worsened, no change). Left panel: VMT eyes without PPV or spontaneous resolution. Middle panel: VMT eyes with spontaneous resolution. Right panel: VMT eyes undergoing PPV. logMAR, logarithm of the minimum angle of resolution; PPV, pars plana vitrectomy; VA, visual acuity; VMT, vitreomacular traction

There were 46 eyes with spontaneous resolution in the VMT group (Fig. 7). The majority of these eyes (69.6 %, 32/46) resolved within 180 days. VA data were available for 41 of these eyes (89 %). A total of 14 eyes (34.2 %) had improved VA at the last observation (Fig. 8, middle panel; *P* = .001). The majority of eyes in the VMT group (105/188) were followed under continued observation, ie, they were not actively treated and did not experience spontaneous resolution. Of these 105 eyes under continued observation, 9.5 % did have improved VA and 7.6 % had decreased VA (Fig. 8, right panel). The median change in VA was not statistically significant (*P* = .97).

In the subgroup of 52 VMT eyes that underwent PPV, VA data were available for 42 eyes (81 %), of which 35.7 % had improved VA and 14.3 % had decreased VA compared to baseline. Although there was a trend to improved VA at the end of the observation period, this was not significant (Fig. 8, left panel; P = .09). Overall, of the 188 eyes with VMT, only 39 (20.7 %) had improved VA at the last observation, whereas 16 (8.5 %) worsened.

#### Complications

No retinal detachments were reported after surgery. No phototoxic effects were observed; note that ILM-Blue does not have phototoxic properties such as those described after using indocyanine green (ICG) for staining the ILM. No late reopenings of macular holes were reported. Since all phakic eyes had lens surgery during the first intervention (all patients were >50 years of age and consented to surgery), no cataract development was reported.

#### Independent predictors of PPV in eyes with VMT

Metamorphopsia increased the odds of patients electing to undergo PPV by a factor of 3.33. Being female increased the odds of undergoing vitrectomy by a factor 2.45 compared to being male. For a difference of 1 ETDRS line of baseline VA, the odds of undergoing PPV decreased by 13 % (odds ratio 0.87) (Table 5).

**Table 5:** Logistical regression model for baseline predictors of vitrectomy in eyes with vitreomacular traction

Variables	Coefficient ± SE	z	P Value	95 % CI	Odds Ratio
Metamorphopsia	1.20 ± 1.23	3.25	.001	1.61, 6.88	3.33
Female	.90 ± 0.91	2.40	.016	1.18, 5.08	2.45
ETDRS lines	−0-.14 ± 0.05	−2.70	.007	0.78, 0.96	.87

*CI*, confidence interval; *ETDRS*, Early Treatment Diabetic Retinopathy Study; *SE*, standard error.

Odds ratio = e^coefficient^

## Discussion

In this retrospective analysis, the burden of tractional VMI disease was found to increase with each progressive stage of the disease (VMA < VMT < MH with VMT < MH without VMT). The presence of metamorphopsia, considered a hallmark symptom of vitreomacular pathology [6–9, 12, 14, 15], correlated with the worsening disease stages. Similarly, worsening VMI disease was associated with increased vision loss. We found metamorphopsia to be a frequent cause for referral to our center. Although metamorphopsia alone was not the indication for vitrectomy, it was one of the key symptoms bringing patients to the clinic, and therefore, was one of the factors considered when choosing PPV. Indeed, the National Institute for Health and Care Excellence (NICE) in the UK has officially stated that having metamorphopsia has the same burden on the quality of life as a 2-line decrease in VA. In our analysis, having metamorphopsia was a strong independent predictor of vitrectomy in eyes with VMT, tripling the odds of a patient opting to undergo PPV. This finding suggests that VA measurement only may not be the most adequate correlate of disease severity.

Our findings underscore that watchful waiting may not be an appropriate course of action, as delayed treatment can lead to further disease progression. In eyes with VMA, 1 in 10 had disease progression and 1 in 20 progressed to a threshold where the patient opted for PPV. A total of 5 % of eyes with VMT developed a new macular hole, almost half in the first month after diagnosis, and the majority of those new macular holes progressed to a threshold where the patient opted for PPV. Moreover, none of the MH with VMT cases showed a spontaneous closure, even if 15.1 % showed a spontaneous VMT release, rendering them unsuitable for treatment with pharmacological vitreolysis that could potentially induce a nonsurgical closure (previously shown in 40 % of MH with VMT [41, 42]). In the VMT patients, only the subgroup showing spontaneous resolution of traction demonstrated an increased VA outcome compared to baseline, whereas this was not the case in the group without VMT release, nor in the group that underwent PPV (where preoperative VA was usually decreased to a level far lower compared to the baseline measurement).

Eyes with a macular hole at presentation (with or without VMT) were recommended for PPV. In our analysis, 199 of 229 eyes with a macular hole underwent PPV (86.9 %), the vast majority within the first 3 months. Thirty patients (13.1 %) with macular holes declined surgery. Only one eye (in a young patient with a post-traumatic macular hole) had a spontaneous closure.

Intraoperative PPV complications are not uncommon, with cataract formation likely in phakic patients [16], which is often a reason to opt for watchful waiting rather than rush patients with milder forms of VMI into PPV.

In this study, a high percentage of patients underwent PPV—more than 85 % in each of the MH groups and more than 25 % in the VMT group. The reported vitrectomy rates could have been even higher since the center was aware of pharmacologic vitreolysis and was enrolling patients in the Safety and Efficacy Study of Microplasmin in for Non-Surgical Treatment of Focal Vitreomacular Adhesion (MIVI-8) study, thereby disqualifying them from inclusion in this analysis.

Others have reported much higher percentages of spontaneous resolution (ranging from 11 % to 53 %), but these series also had far fewer patients [10, 22, 25–30, 34–36]. Larsson et al. [26] reported on 11 patients (11 eyes) with VMT who had deferred PPV for 3 months, and during that time, no cases of spontaneous resolution were observed. Odrobina et al. [27] suggested vitreous surface adhesion and ERM were closely tied to spontaneous resolution, and that a higher spontaneous resolution of VMT may have been the result of fewer ERMs. In our analysis of eyes with VMT, 22.7 % experienced spontaneous resolution, but 5.4 % progressed to a macular hole and for another 25.6 % patients opted for PPV (almost half of them within the first month of diagnosis). One reason patients may opt for PPV is that studies have shown that spontaneous resolution may take a long time to occur (mean follow-ups exceeded 2 years in some studies) and that symptoms may persist or worsen over time even after successful resolution [22, 43].

Our observations further add to the literature on VMT. Although a large proportion of eyes in the VMT group had Snellen VA of 20/40 at time of diagnosis, 55.2 % had been referred for vision loss and 22.2 % for metamorphopsia. Those who underwent PPV registered only a median logMAR change of −0.10, underscoring that VA alone may underestimate disease severity. Of interest, however, is that 14.3 % of eyes with VMT that underwent PPV had a clinically meaningful loss of VA. Of those in the “MH with VMT” group, 92.5 % were diagnosed with metamorphopsia and 54.7 % had been referred for metamorphopsia even though baseline VA was 20/80.

Almost 40 % of all patients had bilateral involvement, which was higher in this group of patients than anticipated. As might be expected, bilateral disease results in substantially more compromised vision than unilateral cases. As this is a progressive disorder with limited success in spontaneous resolution, we recommend continued vigilant follow-up in an affected fellow eye.

Our cohort was followed for a mean of 10.9 months (median 6.9 months). Other studies had longer follow-up times, but observed fewer numbers of patients. For instance, Hikichi et al. [22] followed patients for 60 months with a study of N = 53 eyes. Similarly, Dimopoulos et al. [30] had a median follow-up of 594 days (19.5) months with a cohort of N = 46 patients, and Theodossiadis et al. [34] followed 192 cases with a follow-up of 21.8 ± 10.6 months. Other study follow-up times ranged from none to 15.8 ± 8.4 months [10, 25–29, 31, 32, 35, 36].

This study has several strengths, including that it is the largest cohort of patients and eyes with VMI. Other observational cohorts ranged in size from 11 to 214 [10, 22, 25–36]. Our analysis also included the largest number of subgroups and used the most up-to-date disease classifications, from the International Vitreomacular Traction Study Group [37]. Furthermore, in addition to using the most technologically advanced diagnostics available at the time (SD-OCT) to confirm the diagnosis, the same SD-OCT device was used in all patients during all follow-up visits. The ability of SD-OCT to identify the various stages of VMI at earlier points in time has been previously established [10].

However, this study is not without limitations. As a retrospective study, certain variables such as uniform follow-up visits were not possible to establish. In addition, certain data were not available that would have added to the analysis, such as adhesion size and resolution of metamorphopsia. Patients were enrolled from a single-center tertiary care center, which may have a higher proportion of people with VMI referred for diagnosis and may not be representative of other tertiary care populations. As we noted, the number of PPVs in earlier stages of the disorder may have been subject to investigator bias, as investigators were not proactively counseling patients to undergo PPV, except in patients with a macular hole, where PPV was performed expeditiously. In addition, the lack of regularly scheduled follow-up visits was also a limitation.

The present study (termed ReCoVit) was unable to address which patients who present with VMA or VMT are likely to spontaneously resolve and which will progress, nor does it reveal any evidence of the occurrence of tractional diseases of the VMI in a general population. Additional research is needed to address this question, especially in today’s clinical setting, given the regulatory and commercial availability of pharmacologic vitreolysis that provides an alternative to watchful waiting [42].

In conclusion, this retrospective analysis confirmed that metamorphopsia is a hallmark symptom of vitreomacular pathology, and that both vision loss and metamorphopsia increase with disease stage severity. Our findings suggest there is no clinical benefit in watchful waiting, and the delay in treatment may result in poor visual outcomes even after restoration of the anatomical defect.
